# Upon Some Points in the Etiology of Hereditary Syphilis

**Published:** 1876-06

**Authors:** F. R. Sturgis

**Affiliations:** Clinical Lecturer on Venereal Diseases in the University of the City of New York; 16 W. 32nd St., New York


### UPON SOME POINTS IN THE ETIOLOGY OF HEREDITARY SYPHILIS.

By F. R. STURGIS, M.D.,

Clinical Lecturer on Venereal Diseases in the University of the City of New York.*

Mr. President and Gentlemen of the Association:

In this evening's paper I beg to present, more to elicit a discussion of your views than to offer any original or startling theory, a criticism on the opinion which still obtains among a large part of the medical profession, that syphilis is transmissible to the ovum in utero by the semen of the male parent, without the mother becoming infected either by the husband or by the ovum.

This question has been for a long time a moot point among medical men, and indeed seems as far from settlement now, as it was when enunciated by Cullerier in 1854 before the Société de Chirurgie de Paris, if we regard the papers and monographs written by the upholders of both sides of the argument—those for and those against.

In 1871 and 1873, I wrote two papers criticising and weighing the evidence offered by both sides, and showing why the testimony given in favor of this mode of transmission was defective and unworthy of acceptance. Let me say this at the outset, that those who accept this paternal transmission theory, and seek to prove its truth by cases, enter the race heavily weighted as regards their antagonists; all these latter have to prove is the good health of the mother and child, while the former have to prove: first, the undoubted existence of syphilis in the father and child; second, the non-existence of the disease in the mother, not only in the present but in the past and future; and third, to explain sundry anomalies and contradictions which the other side are not troubled with. Little wonder then, gentlemen, if the absolute proof be difficult to give.

* Read before the Medical Journal Association of the City of New York, May 26, 1876.

In the first place let us examine the question theoretically. Why is it unlikely that syphilis should exempt the mother and attack only the ovum? To do this properly we must explain what syphilis is and how it acts, at least as regards contagion. We believe it to be a disease which, during some of its stages, notably the earlier ones, is eminently contagious, so much so, that, given the proper conditions, it will be propagated to others who are free from the disease, without any respect of person, sex or age. The virus of syphilis has not yet been isolated, perhaps never will be, but by experiment we are in a position to say that similar symptoms will be induced in a non-syphilitic person by the mere fact of bringing in contact with the absorbents the secretions from the chancre (initial lesion), from mucous patches and the corpuscles of the blood during the first year, at least. The serum does not seem endowed with this property. The transmission by the secretions of other lesions is not yet proven ; and infection by the natural excretions of the body, viz. : the tears, sweat, saliva, milk, etc., with but one exception, the semen, have been excluded as impossible. So that in order to accept this theory we must allow that a healthy mother can without danger to herself retain, and perhaps bring to full term, a diseased fœtus, and this child, the moment it comes into the world, becomes the centre of contagion for all non-syphilitic persons who may be unfortunate enough to get the blood or the secretions from the mucous patches of the child in contact with their absorbents, excepting the mother, who, wonderful to relate, escapes all contagion although she suckles the child herself—a fact noted by Mr. Abraham Colles, of Dublin, as early as 1837.

The first point, viz., why the mother should escape the disease while the fœtus is yet in utero, is believed to be explained in this manner, and I quote from the latest writer on the subject, M. Kassowitz, of Vienna, a firm believer in this theory, and whose experience in the repeated immunity of the mother has been so fortunate that, were it borne out by general experience, it would almost settle the point in dispute. He writes: "There is every probability from the facts just detailed, for believing that the syphilitic poison is not transmitted from the mother to the fœtus, provided that at the time of conception the mother was not syphilitic, nor does the child, rendered syphilitic by the father's semen, transmit the disease to the healthy mother, and moreover this virus does not pass through the walls of the vessels between the circulatory apparatus of the mother and fœtus." Thus we see the endosmosis and exosmosis of the virus is denied, and if this theory be the correct one, how can we explain the absorption of the virus in acquired disease? If I understand it correctly, the poison is absorbed from the point of entrance into the mass of the blood. How can this be effected but by endosmose; by the blood carried elsewhere, and deposited in the tissues by exosmose; is it not? There is, then, continued endosmosis and exosmosis going on, else the virus would remain encysted and could work no mischief. Then, again, look at it from the hereditary point of view: syphilitic women give birth to syphilitic babies, where, so far as we know, the husband is free from the disease; if the virus did not transude through the walls of the maternal blood-vessels to poison the fœtal blood, where did the child get its disease? Let me give a case briefly: A woman married a man who contracted syphilis, by him had a child which showed unquestioned syphilis congenita; she herself meanwhile remained healthy, *i. e.*, showed no signs of syphilis. The husband died; she, still being free from syphilitic disease, married a man who was perfectly healthy, and four years after marriage has a syphilitic child. (VIDAL, *Gaz. d. Hop.*, 1841.)

Now how to explain it. The simplest and probably most correct answer would seem to be, the woman's immunity from syphilis was only apparent, she was probably diseased. It may be urged that the second husband was diseased; the history, however, states explicitly that both were free from syphilis, and there is no good reason for accusing him more than her. If we admit the freedom from disease of these two persons, we are driven to admit that healthy, *i. e.*, non-syphilitic, persons can procreate a syphilitic child, a proposition so manifestly absurd as to need no comment.

In former papers, I have given reasons why physicians are likely to be deceived in investigating this question, partly from natural causes dependent upon the disease, and partly from the ignorance and deceit of the patients. It is unnecessary, therefore, to revert to them here, and in arguing against this view I shall confine myself to a consideration of the cases of M. Kassowitz, who is the latest exponent of the doctrine. He takes the records of the K. K. Findelhaus, in Vienna, from 1854 to 1868 (for 15 years,) and states that out of 400 cases of syphilitic children, in 122 the mothers were syphilitic, in 112 the condition of the mother was unknown (mutter unbekannt) and in 166 the mother was healthy. I have been unable to get at the sources of his facts to verify his statements, but allow me to point out one or two things in the table which strike me as curious, to say the least. In the first place, what is meant by saying that the condition of the mother is unknown (mutter unbekannt)? she must have been either ill or well, *i. e.*, showing signs of disease or free from them; if the former, the mother should be ranged under the head of "mother syphilitic;" if the latter, under the head of "mother healthy," or rather, what would be probably more correct, the healthy mothers should be put under the head of "unbekannt."

Again, as the record stands, it is decidedly opposed to the experience of other observers. Oewre, of Christiania, gives the results of 100 syphilitic children watched by him in the University Hospital at Christiania, and they stand: Mothers diseased, 96; in only 4 cases were symptoms absent, although in even these cases, M. Oewre says there was room for question. (OEWRE,

*Aftryck ur Nordiskt Medicinskt Arkiv. Band V, No.* 19.)

O.'s cases were one-quarter the number of Kassowitz'. Suppose we make them equal for purposes of comparison :

| | No. of Syphilitic Children. | Mothers Syphilitic. | Mothers Unknown. | Mothers Sound. |
|---|---|---|---|---|
| Kassowitz........... | 400 | 122 | 112 | 166 |
| Oewre......... ..... | 400 | 384 | .... | 16 |

A very great difference, as you see. M. Kassowitz in his private practice relates 119 cases; of these, the connection between the disease and hereditary transmission was doubtful in 43, exclude them, and we have 76 cases, in 43 of which the mothers were entirely free from syphilis ; in *twenty-three cases both parents*, and in ten the mother only was affected with syphilis.

To condense—

| | | |
|---|---|---|
| Total cases, - - - - - - - | 119 | |
| Doubtful, - - - - - - - - | 43 — | 76 |
| Mother free from syphilis, in cases, - | 43 | |
| Both parents syphilitic, " - - | 23 | |
| Mother alone diseased, " - - | 10 — | 76 |

K. bases the immunity of the mother upon careful examination, for the most part extending over several years, (durch eine umsichtige und in dem allermeisten Fällen durch viele Jahre fortgesebzte Beobachtung.) In this respect he has been more careful than others who have reported upon supposed cases of paternal transmission, but even his length of time is too short. To paraphrase the well-known saying, "Call no man happy till his death" into "Call no person free from syphilis until death," in face of the positive evidence that we have against this theory, we may say of M. Kassowitz' cases that he has not seen or known about the true history of these women.

Indeed we cannot be positive that syphilis will not reappear after many years latency, and I will only remind you of Fournier's case, reported by him in *L'Ecole de Méd.*, Aug. 30, 1875, and quoted in *Lyon Médicale* of Sept. 19, 1875, to show that it does. Seventeen years of latency did not protect the unfortunate patient (who, by the way, was a medical man) from a sudden explosion of nervous syphilis with death. It is for this reason I say, that even though no lesions are visible for four years, the length of time that some of K.'s cases were under inspection, that fact proves nothing, nor does it disprove the possible existence of syphilis.

Of these 43 cases where the mother was free from syphilis, M. Kassowitz gives the history of 10; of the 10, 5 of the mothers were under inspection for a time varying from one to four years without showing symptoms of disease; in the 5th case, the mother is merely spoken of as well (die Frau war und blieb ganz gesund), no history of examination or time she was under inspection; in No. 6, no mention of mother's condition after the birth of syphilitic child; in No. 7, the mother is mentioned as entirely healthy, nothing more; in No. 8, no mention of mother's condition; and of No. 10, I wish to speak a little more fully. It occurred in his private practice, and is headed—

"ADVANCED SYPHILIS IN THE HUSBAND, WIFE HEALTHY. AFTER THREE STILL-BIRTHS MERCURIAL TREATMENT OF THE MAN, BIRTH OF A HEALTHY CHILD. SUBSEQUENT TERTIARY MANIFESTATIONS IN THE FATHER."

"A well-to-do citizen, formerly an officer, contracted primary syphilis in 1864, followed by secondary symptoms, and from which he apparently entirely recovered. In 1867, he married a young lady, 20 years of age, of good family."

"At the end of 1867, she had a still-born boy at the

6th month ; in 1868, a still-born girl at the 7th month, and, 1869, a miscarriage at the 3rd month.

"Externally there was nothing visible upon these children. During the winter of 1872 and 1873, the husband was attacked with serpiginous ulcerations of the skin. He was treated by inunction and subsequently by the iodide of potassium by one of the first syphilographers of the city and his assistants (to whom I am indebted for the history of the father's syphilis), and in the early part of 1873, seemed entirely well. About this time the wife conceived for the fourth time, and in January, 1874, was brought to bed at full term of a healthy, unusually strong girl child. Shortly after birth, the child showed symptoms of dyspepsia (it had been artificially reared), came under my care, and from that time to the present has been under constant supervision. It has never shown a symptom of inherited syphilis. In the summer of 1873, the father went to the iodine baths at Hall, notwithstanding which he was attacked during the following winter with gummata of the skin and necrosis of the nasal and ethmoid bones. In the spring and summer of 1874, he was sent back to Hall, where he died in midsummer of the same year with the symptoms of miliary tuberculosis."

"The wife who had never been informed of the nature of the husband's disease, and is to-day ignorant of the cause of her miscarriages, being pronounced by the attending physicians as healthy, was naturally never put upon an antisyphilitic treatment, and has for the past two years, during which I have had repeated opportunities for observing her, enjoyed the most perfect health."

At the end of the narration of the history, the author exclaims, "this case also, by the birth of a healthy child, demonstrates in the most striking manner the freedom of the mother from syphilis," to which I answer, Amen. But that is not the point; the argument is, whether syphilis has not been conveyed to the ovum by the semen, and in this case upon what does this rest? Upon three miscarriages without any syphilitic symptoms. I object to that as evidence; miscarriages occur from other causes than syphilis, and their occurrence *alone* is not sufficient to base a diagnosis of syphilis per seminem. And M. Kassowitz is evidently of the same opinion, for on p. 54, when discussing the means to be employed for the exclusion of error, he lays down three rules to be observed, the third of which reads: "The syphilis of the child must be evident from undoubted symptoms. Premature births, death in utero, a sickly constitution at the time of birth, speedy death without any preceding outbreak of syphilis, are not by any means sufficient for founding a diagnosis of syphilis in the child."

If we act upon this instruction how can we admit the existence of syphilis in any one but the father in the case just cited? The case must be thrown aside as far as proof is concerned; indeed, were it used at all it would rather tend to show that a syphilitic man can procreate a healthy child, whereas the opposite should obtain, if the paternal transmission theory be correct.

I mentioned in an earlier portion of the paper the curious fact, that mothers apparently free from syphilis are able to nurse their own babies which are syphilitic and eminently contagious, without contracting disease, where strange women performing similar acts, usually pay the penalty of their rashness. Kassowitz also acknowledges that he has never seen infection from such a cause, and then goes on to say: "It would be proper for those who regard every mother of children syphilitic by inheritance, as the subject of latent disease, even though she present no evidence of syphilis, to prove the correctness of their view by experimental inoculation." This very condition was fulfilled in one case by Caspary in the 4 Heft der Vierteljahrschrift für Dermatologie und Syphilis for 1875. Although this one case does not absolutely prove anything, inasmuch as the result may be considered as coincidental, combined with other evidence it becomes strongly confirmatory. I shall be obliged to give the case in a somewhat condensed form for want of space and time.

*Father.* Undoubted syphilis ; primary lesion in 1872, several attacks of skin lesions ; obstinate iritis and various nervous symptoms.

*Mother.* Nothing which could be laid to syphilis, had been married for several years prior to husband's infection and had had healthy children. In 1874, became pregnant and aborted at third month.

*Fœtus.* At the third month ; umbilical cord entirely macerated ; of a dirty gray color ; that and the inner surface of the cavities of the body, together with the muscular tissues, were undergoing fatty degeneration. The placenta was in some places thick and fibrous ; in one part was soft and spongy—under the microscope it showed fatty degeneration.

The mother still showing nothing, Caspary persuaded her to be inoculated, which was done, "upon the left arm in four places with the secretion taken from condylomata lata, mixed with blood. The person from whom the secretion and blood were taken, was in the beginning of the eruptive stage of syphilis and had never been treated. The result was negative, and after waiting for six weeks without anything appearing, the woman was put upon treatment."

Here is a curious case : a woman apparently free from syphilis gives birth to a three months fœtus, which does not, it is true, present indubitable signs of syphilis. Were this all, the question might very properly be raised, are the mother and fœtus diseased ? but what shall we say, when we find that inoculation utterly fails upon the mother ? I believe we are taught that the only thing which gives immunity to acquired syphilis is a previous attack of the same disease. If this be true, we must conclude that this woman was syphilitic, notwithstanding her apparent good health, and this evidently was Caspary's belief also.

Interesting as the examination of such cases are, my time warns me that I cannot devote more space to this class, if I am to review the subject where syphilitic fathers have healthy children. Before doing so, however, let me give one case from the late Mr. Langston Parker's little work "*On the Mercurial Vapor Bath*," as it is peculiarly instructive.

"A young gentleman and lady married, with all the prospects of future happiness that fortune and apparent health could give. In due course the lady became pregnant, but miscarried. The same thing happened in her second and third pregnancies; a good deal of mental uneasiness was produced, and some suspicions arose. The fourth child was born alive, but at six weeks old had snuffling and the eyes became bad; condylomata also appeared about the arms. A neighboring physician of great local eminence was consulted, who said rather abruptly, 'The child is diseased.' The parents, as may naturally be supposed, were shocked and horrified beyond measure, the father having at a remote period before his marriage been affected with syphilis; but the mother had never exhibited the least symptom of the disease. He was put upon a course of blue pill and iodide of potassium; the mother at first was not treated. A fifth child was born, who at the end of the first month had symptoms of syphilis. The father was again only treated, and a sixth child was again born diseased. The mother was once more examined, but no trace of the disease could be found in the throat, vagina, uterus, or elsewhere. The patients were now placed under my care. I recommended that both should be treated by a full course of mercurial vapor, and that no intercourse should take place during that period. The seventh child was born healthy, and has remained so, and neither father nor mother have as yet exhibited any further symptoms of disease."

What I wish to call attention to in this case is, that though the father who was considered the sole cause of the children's disease was repeatedly treated, the children persisted in being syphilitic contrary to what ought to have been the case, and it is not until the mother, who, mark you, was reputed free from syphilis, is subjected to antisyphilitic treatment, that this persistence is broken. If the disease came from the father alone, why was treatment in him so inefficacious? It is hardly explicable, unless we believe the mother's good health was only apparent, not real.

On turning to the other side of the question, where syphilitic fathers have healthy children, so long as the mothers escape infection, we are at once struck with the straight forwardness of the evidence and the regularity with which the phenomena occur. It is not a new opinion, broached for the first time by Cullerier, although he it was who in recent times has brought it more prominently forward. Hunter, as far back as 1786, in his *Treatise on the Venereal Disease*, writes: "Hence it has been supposed that the testicles and vesiculæ seminales may be affected by the disease; that the semen may become venereal, may communicate the disease to others, and after impregnation may even grow into a pocky child. But this is all without foundation." * * * *

These views, however, were not generally accepted, and Cullerier, in 1854, before the Société de Chirurgie of Paris, stated that he had met with cases which shook his faith in the belief that syphilitic fathers must have syphilitic children, and he furthermore expressed his opinion that so long as the mothers escaped infection the children would be born healthy, and that the paternal disease had no direct influence upon the children's health. He then gives two cases which I here give in a condensed form:

1. *Father.* Indurated chancre, mucous patches of the anus, ulceration of the mouth, impetigo of the scalp, alopecia and cervical adenitis. After 15 days treatment, salivation. Notwithstanding all this, he married.

*Mother.* Showed no symptoms of syphilis either then or afterwards.

*Child.* Entirely free from syphilis. Age at time of reporting case, 18 years. Two children born later, also healthy.

2. *Father.* Indurated chancre six months before marriage ; subsequent symptoms : roseola, cervical adenitis, and trouble in throat. During this condition of the husband the wife became pregnant.

*Wife.* Never showed symptoms of syphilis.

*Child.* The same as mother. Age 15 years.

M. Notta in 1860, published in the *Archives Générales de Médécine* his observations with the results, which amount to 11 in all. Of this number eight of the mothers were not infected ; the number of children born to these eight mothers amounted to 15 : every one of these 15 children was born healthy, and their ages at the time of reporting the cases, ranged from seven months for the youngest to 12 years for the eldest. Three mothers are left to account for : to these three, five children were born : the mothers were all syphilitic, and so were all the children but one, which died at the sixth month of intra-uterine life.

M. Charrier follows in the same journal with seven cases : in five of these, the mothers were entirely free from syphilis ; the number of children born were nine, and all were healthy. The ages of these children ranged from eight months to six years. Of the remaining two cases, the mothers were infected ; the number of children was five ; one died a month after birth syphilitic, two were miscarriages, one at the fourth, the other at the seventh month, the latter with mucous patches at the anus, and two were abortions at the third month, both the fœtus covered with copper-colored spots.

I need hardly mention that in all these cases the fathers were unquestionably syphilitic.

Now let me call your attention to one case, the second of M. Charrier's list. The man had a wife and a mistress.

*Husband.* Palmar syphilide.

*Wife.* Mucous patches of the anus, with subsequent lesions not detailed.

*Mistress.* Perfectly healthy—not the slightest signs of syphilis.

*Children*, (by the wife). One born healthy. Twenty days later; mucous patches, emaciation. Death one month after birth. This was in 1855. In 1856, a miscarriage at the fourth month, and again in 1858, at the seventh month. In this latter the child had mucous patches at the anus.

*Children*, (by the mistress). One, which never showed the least sign of syphilis: age, three years. This birth occurred within fifteen days of the wife's accouchement in 1858.

Objection may be raised as to this last child really being the man's: M. Charrier also considers this objection, and writes, "Mais j'ai à répondre que cet enfant ressemble en tout point à son père et qu'il a comme lui une conformation toute particulière des pouces, que les enfants légitimes avaient également présentés."

Diday, of Lyons, also gives a case:

*Father.* Undoubtedly syphilitic.

*Mother.* Perfectly healthy.

*Child.* Syphilitic? Oh no! Never showed the faintest sign of syphilis, although it was two years old at the time of reporting the case.

Mireur also gives two cases equally conclusive, one of them so apt and to the point that I will give it in detail.

"In January, 1863, M. C., employed in a government office, contracted an indurated chancre situated in the balano-preputial furrow, which was speedily followed by a double inguinal adenitis. By the advice of his physician, M. C. began mercurial treatment at once. This was followed for five weeks, when secondary symptoms supervened: a papulo-macular erythema, ulcerations of the throat, and impetigo capitis. In consequence of the energetic treatment to which the patient was subjected, one lasting for over four months, all the constitutional symptoms disappeared.

"Nothing having appeared up to December of the same year, M. C. believed himself to be entirely cured. Notwithstanding the advice of the surgeon who attended him, M. C. determined to carry out a project of marriage which he had had in mind for some time.

"Immediately after marriage, Mme. C. became pregnant. In October, 1864, Mme. C. was brought to bed of a very fine boy, perfectly healthy, and of a good constitution.

"This child, the image of his father, grew up and was perfectly healthy to the age of two years. Towards the end of 1866, M. C., who retained a few vague souvenirs of his former disease, and who was charmed with the admirable health of his boy, had on the anterior portion of the lower lip, a slight erosion. As it remained perfectly indolent he thought nothing of it, and continued to kiss his child just the same.

"A short time after, the child showed upon his lip an erosion with a depressed surface, of a livid tint, with a remarkably indurated base, one centimètre in diameter. There was no doubt of its being an infecting chancre. Indeed, a short time after, a maxillary adenitis, with roseola syphilitica and extensive mucous patches of the anus, developed themselves."

Comment is scarcely necessary. If we accept the old theory this child should have been syphilitic and therefore not obnoxious to the action of the virus—but what do we find? Born healthy and remaining so up to two years of age, notwithstanding his father's syphilis, he becomes subsequently the subject of acquired syphilis.

Mireur's second case is briefly this:

*Father.* Primary lesion. Erythema syph. and ulcerations of the throat.

*Mother.* Never syphilitic. Strong and well.

*Child.* Never syphilitic—age 13 years.

Father subsequently had other syphilitic symptoms.

Oewre, of Christiania, has also presented several facts bearing on this question. In 1872, he gave the history of 24 fathers, all of whom were syphilitic. These 24

fathers have 42 children: not one syphilitic. In 1873 these numbers became: 29 fathers, 55 children, same results, not a syphilitic child.

As personal experience is always the most convincing to any to whom it occurs, allow me, in concluding this paper, to present three cases which have come under my personal supervision.

The first one occurred in the person of a friend, a college though not a class mate, who contracted syphilis. He was under my care for mucous patches of the tongue and throat, and occasional papular eruptions of the skin. Under treatment, he seemingly recovered and married. His first child was born abroad, and within a few months after birth died of a bronchial affection, without showing any symptoms of infantile syphilis. His statement is, that the attending physician upon being told of his previous syphilis, inclined to attribute the cause of the child's death to that. He was not sensible of having had, since he had been under treatment, any lesion which could be referred to syphilis, nor did he think his wife had.

Upon their return to this country Mrs. X. became pregnant and he wished me to take charge. I examined both carefully; he presented no symptoms. Mrs. X. had always enjoyed good health with the exception of being subject to pharyngitis and chronic inflammation of the middle ear since her girlhood. She presented nothing either from history or examination except a few irritated granular spots in the throat, referable more to pharyngitis, I thought, than anything else. As the husband was extremely anxious, and naturally desirous of taking all precautions, the wife during the period of gestation was placed upon a mild mercurial course. The child was carried to full time and born sound and healthy, weighing between seven and eight pounds. This girl was for some months under observation, but no signs of congenital syphilis were seen. I had occasion to see her four months ago and found her a stout, robust child, four years of age, without a symptom of syphilis, and I am told she has never shown any. The husband during the wife's pregnancy had one or two little mucous patches (?) on the gums and end of the tongue, which disappeared on cessation from smoking and local use of the sulphate of copper. Neither then nor subsequently had he been placed upon mercurial treatment: purposely so.

After confinement the wife's treatment was discontinued; she was kept under observation; nothing was found in either herself or the child.

The family moved out of town, and two years later, no treatment having been pursued meantime, the wife again became pregnant. This third child I saw also four months ago, a fine chubby girl two years of age, showed nothing syphilitic, nor did the mother. The father I did not see, so am unable to tell if he has had any symptoms of his former trouble.

It occurred to me, if the father's semen was at fault, why were the children born healthy? He had syphilis, showed some signs of it during his wife's second pregnancy, and was not treated. From the evidence, I am forced to conclude that mother and children both escaped infection, notwithstanding his disease.

The second case was an ex-officer in the regular army whom I treated for chancre, erythema and mucous patches of the throat, mouth and tongue. After several relapses he married; and his wife was delivered of a fine boy whom I carefully watched for two years. In neither mother nor boy could I ever discover symptoms of syphilis, neither was there any history. During the wife's pregnancy and after the boy's birth, the father was under my care for repeated attacks of mucous patches of the tongue and mouth, and a desquamating papulo-erythematous eruption on the palms of the hands.

The third case was a man treated by me for erythema maculatum syph. and repeated attacks of mucous patches of the throat and tongue. He married, and in time and in due time his wife became pregnant and was delivered of a female child, who, when I first saw it, was three years of age. Neither it nor the mother have presented the least trace or sign of syphilis.

After his wife's confinement the father came under my care for an ulcerating tubercle of the leg, which healed promptly under antisyphilitic treatment.

I have endeavored in the foregoing to give a fair idea of how this question stands, a question very difficult of solution. I trust, however, that the criticisms elicited here to-night by you, gentlemen, will throw some light upon this knotty and important problem.

16 W. 32ND ST., NEW YORK.

